# Statin-Associated Immune-Mediated Necrotizing Myopathy: An Underrecognised Cause of Progressive Muscle Weakness in Primary Care

**DOI:** 10.7759/cureus.105830

**Published:** 2026-03-25

**Authors:** Jee Tat Ong, Teh Rohaila Jamil, Say Yee Loo

**Affiliations:** 1 Family Medicine, Universiti Kebangsaan Malaysia Medical Centre, Kuala Lumpur , MYS; 2 Family Medicine, Hospital Canselor Tuanku Muhriz UKM, Kuala Lumpur, MYS

**Keywords:** autoimmune diseases, hydroxymethylglutaryl-coa reductase inhibitors, myositis, primary health care, statin

## Abstract

Statins are among the most frequently prescribed agents in primary care for lipid control and cardiovascular risk reduction. Although generally safe, they are associated with muscle-related adverse effects, ranging from myalgia and self-limiting myopathy to the rare but clinically significant immune-mediated necrotizing myopathy (IMNM). We report a 52-year-old congenitally deaf and mute Chinese man with diabetes, hypertension, and dyslipidaemia who had been on atorvastatin since 2020 and presented with progressive proximal muscle weakness over one year, more prominent in the lower limbs, accompanied by unintentional weight loss. Communication barriers and the absence of classical features of rhabdomyolysis contributed to delayed recognition of an underlying inflammatory myopathy. Laboratory investigations revealed markedly elevated creatine kinase levels, and muscle biopsy confirmed IMNM with strong anti-HMG-CoA (anti-3-hydroxy-3-methylglutaryl-coenzyme A) reductase antibody positivity. The patient responded well to high-dose corticosteroids, with recovery of muscle strength and normalisation of enzyme levels. This case highlights a diagnostic challenge in primary care, where statin-induced myopathy is a common consideration, while statin-associated IMNM remains underrecognised in clinical practice.

## Introduction

Over the past four decades, statins have become the cornerstone of dyslipidaemia management and cardiovascular disease prevention worldwide. Although generally well tolerated, muscle toxicity remains the most common adverse effect encountered in clinical practice, with a reported incidence ranging from approximately 1.5% to 26%, depending on study populations and definitions used. These effects are typically mild and resolve following drug withdrawal [1]. A rare but clinically significant complication is statin-associated immune-mediated necrotizing myopathy (IMNM). This condition is characterised by progressive proximal muscle weakness, markedly elevated creatine kinase (CK) levels, and histopathological findings of muscle fibre necrosis with minimal inflammatory infiltrates [2]. Unlike statin-induced myopathy, IMNM may persist or even worsen despite discontinuation of statin therapy and often necessitates immunosuppressive treatment [3]. It is strongly associated with the presence of anti-HMG-CoA reductase antibodies [4]. Although uncommon, IMNM has an estimated incidence of two to three cases per 100,000 statin users, and the number of reported cases has been increasing globally. However, data from Southeast Asia, including Malaysia, remain limited [5]. Recognition of IMNM can be particularly challenging in patients with communication barriers, as illustrated in this case of a congenitally deaf and mute patient. In such scenarios, careful clinical evaluation, supported by appropriate laboratory investigations, is essential to avoid diagnostic delay. We present a case of statin-associated IMNM encountered in primary care that highlights these diagnostic challenges, further complicated by statin reintroduction and communication limitations. This report emphasises the importance of early recognition and thorough medication review to optimise patient outcomes.

## Case presentation

A 52-year-old Chinese man with congenital deaf-mutism was under primary care follow-up for type 2 diabetes mellitus, hypertension, dyslipidaemia, and gastro-oesophageal reflux disease. He had been prescribed atorvastatin 20 mg daily since 2020 for primary prevention of cardiovascular disease in view of multiple cardiometabolic risk factors. In May 2024, routine blood monitoring revealed deranged liver function tests, with alanine transaminase (ALT) of 277 U/L, aspartate transaminase (AST) of 320 U/L, and total bilirubin of 26 µmol/L. Atorvastatin was withheld at that point, and the abnormal liver function tests were initially attributed to underlying gallstone disease. He subsequently underwent laparoscopic cholecystectomy in June 2024 for symptomatic cholelithiasis. After surgery, his liver function improved, and atorvastatin was restarted at the same dose in August 2024. Throughout the year, he experienced progressive unintentional weight loss from 70 kg to 57 kg, accompanied by gradually worsening symmetrical proximal muscle weakness that became more prominent following reintroduction of the statin. The weakness was most pronounced in the lower limbs, affecting his ability to rise from a chair, squat, and climb stairs. However, he did not report muscle pain, dark urine, or other systemic symptoms. Because of his deaf-mutism, these problems were not recognised until his daily activities were clearly affected.

On examination, he was alert, well nourished, and haemodynamically stable. Muscle bulk appeared preserved. Power testing showed Medical Research Council (MRC) grade 2/5 in hip flexion and abduction, 3/5 in knee flexion, and 4/5 in shoulder abduction, while muscle strength in other groups was normal. Reflexes and sensation were intact, plantar responses were downgoing, and there were no rashes, joint abnormalities, or cerebellar signs. Blood investigations revealed a markedly elevated CK level of 9,989 U/L, with mildly elevated liver enzymes. Renal function was normal, and urinalysis showed no haematuria. The erythrocyte sedimentation rate (ESR) was elevated at 51 mm/hr, while C-reactive protein (CRP) and thyroid function tests were within normal limits. He was referred to the hospital for further evaluation with a working diagnosis of polymyositis. However, other differential diagnoses were also considered within the spectrum of idiopathic inflammatory myopathies, including IMNM, dermatomyositis, and inclusion body myositis. In view of his long-term statin exposure, statin-associated myopathy was also considered an important possibility.

**Figure 1:**
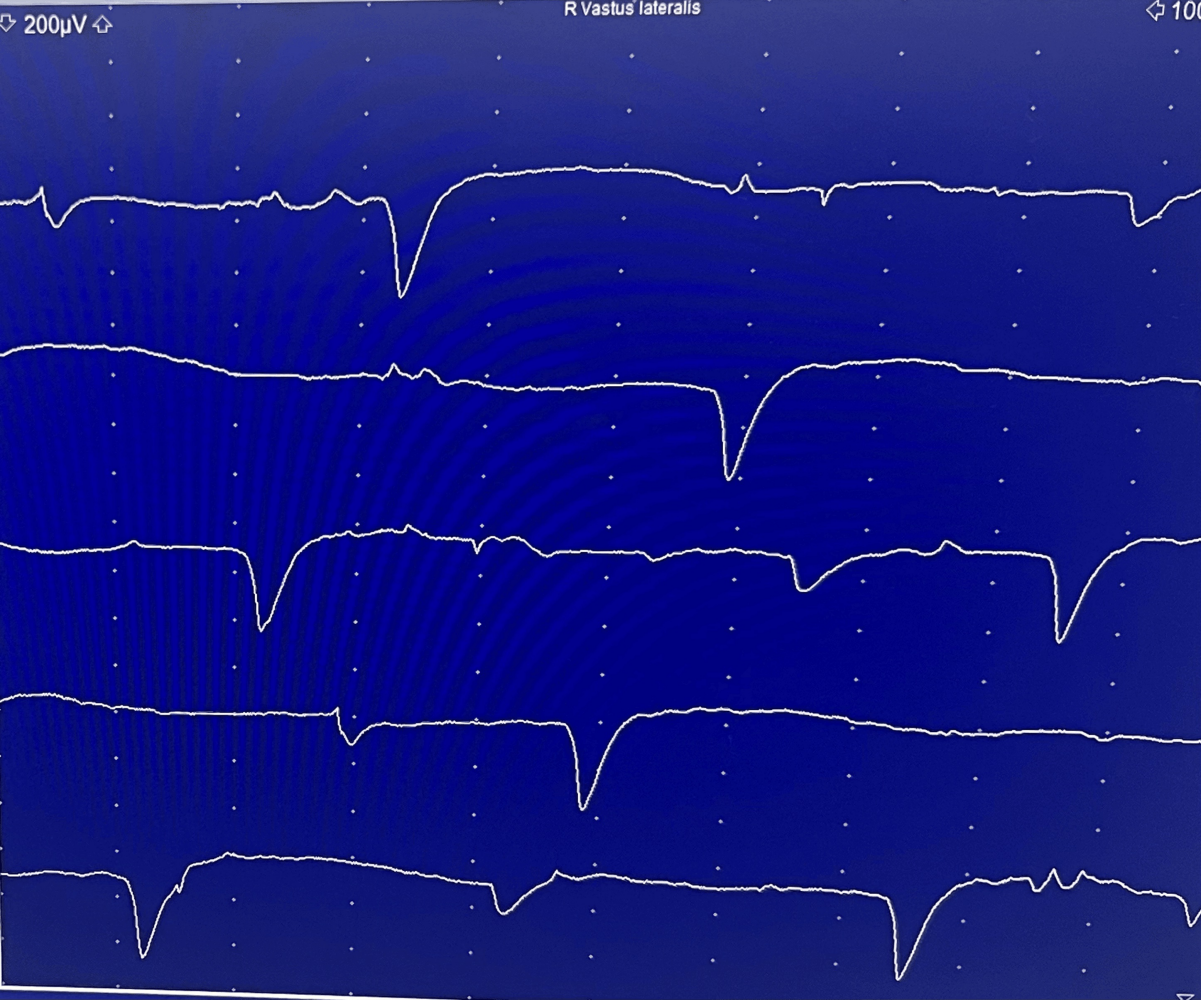
Electromyography of the right vastus lateralis demonstrating myopathic motor unit potentials Needle electromyography of the right vastus lateralis muscle demonstrating short-duration, low-amplitude motor unit action potentials, consistent with a myopathic pattern.

**Figure 2:**
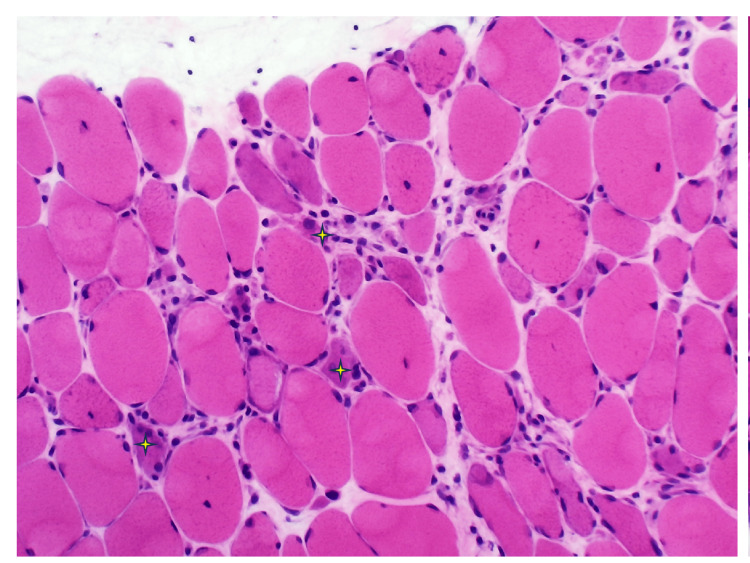
Muscle biopsy (H&E stain, ×200) Hematoxylin and eosin staining demonstrating marked variation in muscle fibre size with scattered necrotic fibres (white four-pointed star).

**Figure 3:**
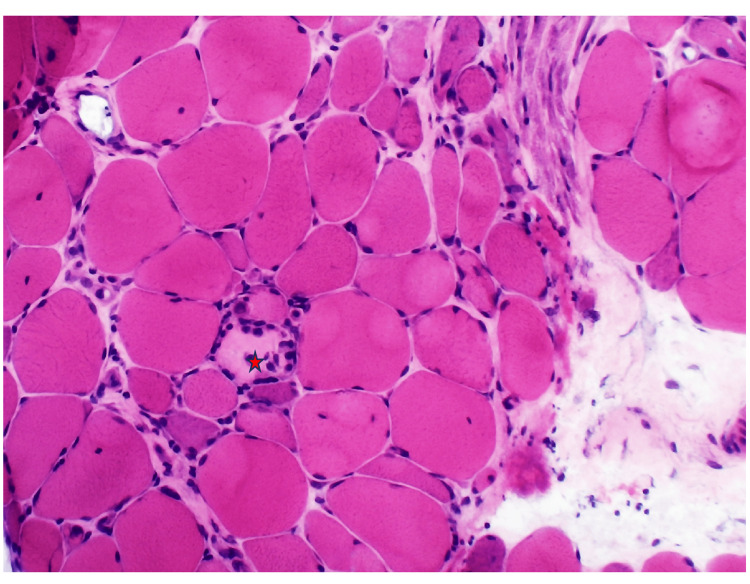
Muscle biopsy (H&E stain, ×200) Hematoxylin and eosin staining demonstrating marked variation in muscle fibre size with scattered necrotic fibres; necrotic fibres are indicated by the red star.

**Figure 4:**
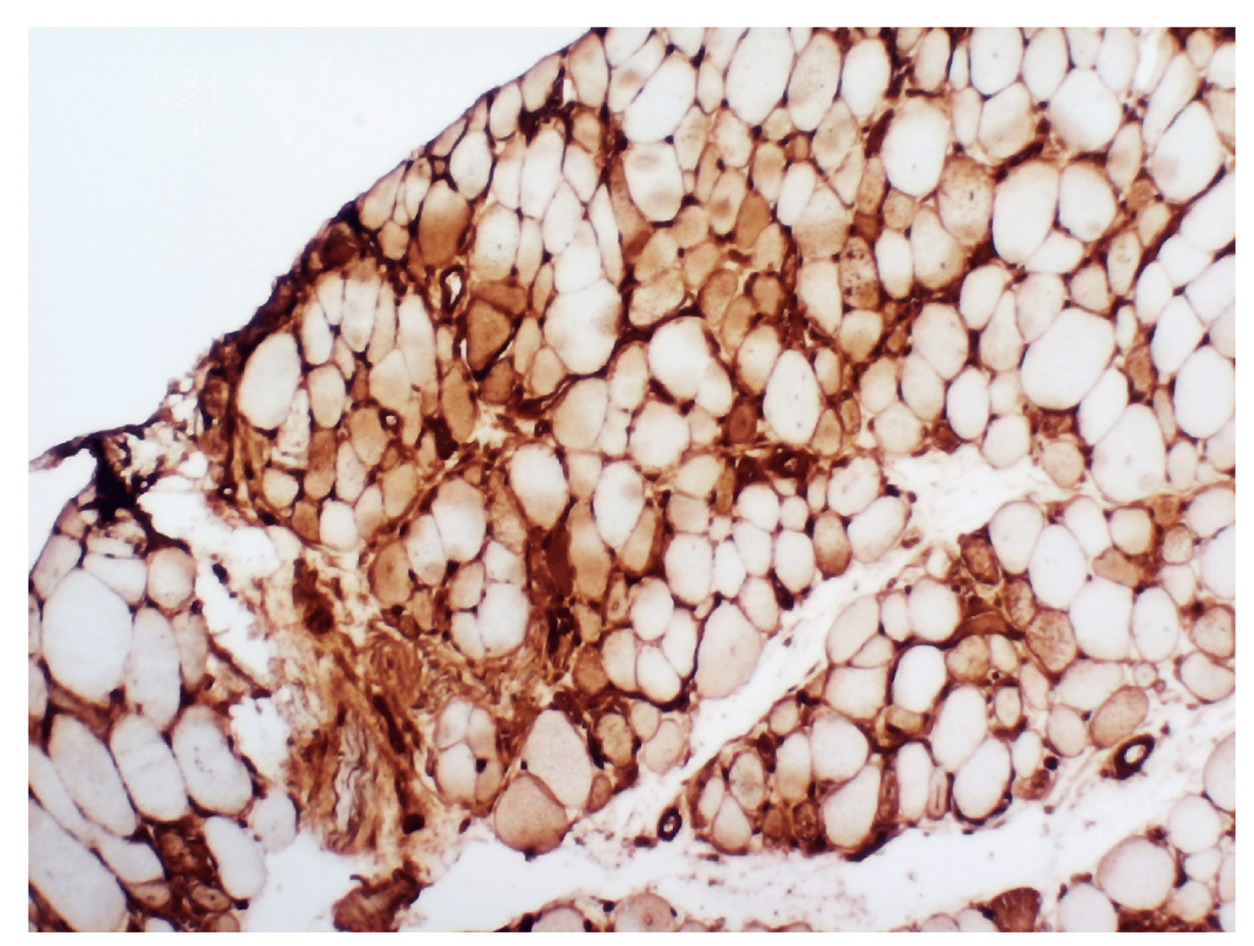
Immunohistochemical staining for MHC-I (HLA-ABC), ×200 Scattered muscle fibres demonstrate moderate to strong sarcoplasmic expression of MHC-I. MHC-I: major histocompatibility complex class I, HLA-ABC: human leukocyte antigen A, B, and C.

In the hospital, autoimmune screening, including antinuclear antibody (ANA), anti-double-stranded DNA (anti-dsDNA), and an extractable nuclear antigen panel (anti-Jo-1, Mi-2, SRP, U1-snRNP, SSA/Ro, SSB/La, and PM-Scl), was negative. Electromyography demonstrated an irritable myopathy characterised by fibrillation potentials and myopathic motor unit action potentials, suggestive of an inflammatory myopathy (Figure 1). A muscle biopsy from the right deltoid muscle revealed marked variation in muscle fibre size, with scattered necrotic and regenerating fibres and minimal inflammatory infiltrates on hematoxylin and eosin (H&E) staining (Figures 2, 3). Immunohistochemical staining for major histocompatibility complex class I (MHC-I; HLA-ABC) demonstrated moderate to strong sarcoplasmic expression in scattered muscle fibres (Figure 4). Overall, these histopathological findings were consistent with immune-mediated necrotizing myopathy. A myositis antibody panel subsequently confirmed strong anti-HMG-CoA reductase (anti-HMGCR) antibody positivity, establishing the diagnosis of statin-associated immune-mediated necrotizing myopathy.

The patient was treated with intravenous methylprednisolone 500 mg daily for five days, followed by oral prednisolone 55 mg daily with gradual tapering of 5 mg weekly. Statins were permanently discontinued, and ezetimibe 10 mg nightly was prescribed for lipid control. At discharge, he was able to ambulate with minimal assistance. Three months later, during neurology follow-up, his CK level had improved to approximately 1,000 U/L, and neurological examination demonstrated full muscle strength (MRC 5/5). He regained the ability to squat and stand unaided, which he had previously been unable to perform. Methotrexate was subsequently introduced as a steroid-sparing agent, starting at 5 mg weekly with plans for gradual dose escalation. Biochemical investigation trends during follow-up are summarised in Table 1.

**Table 1:** Biochemical investigation trends during follow-up “–” indicates value not recorded.

Investigations	Baseline 2020	4 months before admission	On admission	Upon discharge	3 months after discharge	Normal values
Total bilirubin level (µmol/L)	14.5	14.7	20.6	11.6	11.1	3.4–20.5
Aspartate transferase (AST) (U/L)	–	117	206	35	–	5–34
Alanine transaminase (ALT) (U/L)	34	163	92	85	35	0–55
Creatine kinase (CK) (U/L)	–	–	10,139	2,507	1,340	30–200

## Discussion

This case highlights the diagnostic challenges of IMNM in primary care. Muscle complaints in statin users are often attributed to dose-dependent, self-limiting statin-induced myopathy, which typically presents with myalgia, mild CK elevation, and resolution after drug withdrawal [6]. In contrast, IMNM is a distinct autoimmune condition that persists or worsens despite statin cessation, requires immunosuppressive therapy, and is strongly associated with anti-HMG-CoA reductase (anti-HMGCR) antibodies [7]. Diagnosis of IMNM requires a multimodal approach. Clinically, patients develop progressive symmetrical proximal muscle weakness, often severe enough to impair daily activities. Laboratory findings typically include CK levels greater than ten times the upper limit of normal. Electromyography shows an irritable myopathy with fibrillation potentials, while muscle biopsy demonstrates necrotic and regenerating fibres with minimal inflammatory infiltrates, distinguishing IMNM from polymyositis or dermatomyositis [2-4]. The presence of anti-HMGCR antibodies provides highly specific confirmation. In our patient, all these criteria were met, confirming statin-associated IMNM. Although IMNM is rare, its recognition is clinically important. Population-based studies report an incidence of approximately 8.3 per million person-years and a prevalence of 1.85 per 100,000 adults aged over 50 years [8]. Data from Southeast Asia are limited but suggest that IMNM may be more common than previously thought; in a Malaysian tertiary centre, it accounted for nearly one-quarter of all inflammatory myopathy cases, with about 20% being anti-HMGCR positive and therefore statin-associated [9].

The delay in diagnosis in this case was likely multifactorial. Persistent weakness and weight loss were initially attributed to gallstone disease, and statin therapy was reintroduced despite ongoing clinical decline. The absence of myalgia further reduced clinical suspicion, even though up to one-third of IMNM patients present with weakness alone [3,7]. CK testing was delayed until admission, although current consensus recommendations advise CK evaluation in any patient with unexplained proximal weakness, particularly those receiving statin therapy or with prior intolerance [10]. Earlier testing might have expedited diagnosis and treatment.

Communication barriers related to congenital deaf-mutism also contributed, as symptoms were not recognised until his functional ability was significantly impaired. In patients with communication disabilities, diagnostic evaluation may be further complicated by difficulties in accurately eliciting symptoms and assessing disease progression. Previous studies have highlighted that individuals with hearing impairment are at increased risk of delayed diagnosis and suboptimal clinical outcomes due to reduced healthcare accessibility and communication challenges. In such populations, clinicians must rely more heavily on objective clinical findings, functional assessment, and laboratory investigations to guide diagnosis. This case reinforces the importance of adopting a proactive and structured clinical approach, particularly in patients with communication limitations, to ensure early recognition of potentially reversible conditions such as IMNM. In such contexts, simple bedside functional assessments, such as observing the ability to rise from a chair or squat, can provide important diagnostic clues. The differential diagnoses that should be considered for this patient and the relevant features are summarised in Table 2.

**Table 2:** Clinical features and distinguishing characteristics of idiopathic inflammatory myopathies This table has been adapted from Malik et al. [12], which is an open-access article distributed under the terms and conditions of the Creative Commons Attribution License (CC BY). PM: polymyositis, DM: dermatomyositis, IBM: inclusion body myositis, IMNM: immune-mediated necrotizing myopathy, ILD: interstitial lung disease, CK: creatine kinase, ULN: upper limit of normal, EMG: electromyography, CD8: cluster of differentiation 8, MAC: membrane attack complex, HMGCR: 3-hydroxy-3-methylglutaryl-coenzyme A reductase, SRP: signal recognition particle.

Feature	Polymyositis (PM)	Dermatomyositis (DM)	Inclusion Body Myositis (IBM)	Immune-Mediated Necrotizing Myopathy (IMNM)
Demographics	Female predominance, age >40 years	Female predominance, age >40 years	Male predominance, age >40 years	Female predominance, typically middle-aged to older adults
Onset	Subacute, weeks–months	Subacute, weeks–months	Slowly progressive, years	Subacute or rapidly progressive
Weakness pattern	Symmetrical, proximal	Symmetrical, proximal	Asymmetrical, distal > proximal (esp. finger flexors, quadriceps)	Symmetrical, proximal, severe
Extra-muscular features	Rare	Characteristic skin rashes (heliotrope, Gottron’s papules), ILD, malignancy association	Dysphagia common, minimal systemic features	May have dysphagia; often no systemic features
CK levels	Elevated (often 5–50× ULN)	Elevated (5–50× ULN)	Normal or mildly elevated	Very high (often >10,000 U/L)
EMG	Myopathic changes with fibrillations	Myopathic changes	Mixed myopathic & neurogenic	Irritable myopathy with fibrillations
Muscle biopsy	CD8+ T-cell cytotoxicity; macrophage invasion	MAC deposition in microvasculature	CD8+ T cells with amyloid/tau accumulation	Necrotic fibres with macrophage invasion
Autoantibodies	Anti-synthetase (Jo-1, PL-7, PL-12)	Mi-2, TIF1-γ, MDA5	No specific antibody	Anti-HMGCR, Anti-SRP
Treatment response	Steroids + immunosuppressants	Steroids + immunosuppressants	Poor response	Steroids + early immunosuppressants

Management of IMNM differs fundamentally from statin-induced myopathy. The 2017 European Neuromuscular Centre Workshop recommends induction therapy with oral corticosteroids (1 mg/kg/day) or intravenous methylprednisolone (0.5-1 g/day for three to five days) in severe cases, followed within one month by a second immunosuppressive agent such as methotrexate, azathioprine, mycophenolate mofetil, rituximab, or intravenous immunoglobulin (IVIG, 2 g/kg monthly for three to six cycles) [4]. If the response remains inadequate after six months, rituximab may be reconsidered. For maintenance therapy, corticosteroids should be tapered to the lowest effective dose, with methotrexate or rituximab continued for at least two years of remission; IVIG can be reduced as tolerated. Our patient’s treatment included intravenous methylprednisolone followed by tapering oral prednisolone and methotrexate, and this regimen was consistent with these recommendations. His clinical and biochemical improvement supports the effectiveness of guideline-based therapy, even when the diagnosis is delayed. For lipid management, ezetimibe was introduced as a safer alternative, avoiding further statin exposure.

From a regional perspective, IMNM remains underreported in Southeast Asia despite widespread statin use [11]. Greater awareness among family physicians is crucial to identify red flags, request timely CK testing, and initiate early referral. Prompt recognition allows effective immunosuppressive therapy, prevents disability, and improves long-term outcomes.

## Conclusions

This case highlights the importance of maintaining a high index of suspicion in primary care when managing patients on long-term statin therapy, particularly those with communication barriers. In this patient, progressive proximal muscle weakness was not initially recognised, contributing to the delayed diagnosis of statin-associated IMNM, a potentially reversible condition with appropriate treatment. Progressive weakness should not be attributed solely to comorbidities or presumed to represent self-limiting statin myopathy, especially when symptoms persist or worsen despite statin discontinuation. Family physicians play a pivotal role in identifying red flags such as unexplained weakness or weight loss, performing timely CK testing, and carefully reviewing medication history, particularly prior to statin reintroduction. Early consideration of IMNM in the differential diagnosis and prompt referral for specialist evaluation are essential to enable timely initiation of immunosuppressive therapy, reduce the risk of long-term disability, and optimise patient outcomes.
